# Addition of 100 mg of Tramadol to 40 mL of 0.5% Ropivacaine for Interscalene Brachial Plexus Block Improves Postoperative Analgesia in Patients Undergoing Shoulder Surgeries as Compared to Ropivacaine Alone—A Randomized Controlled Study

**DOI:** 10.3390/medicina55070399

**Published:** 2019-07-23

**Authors:** Eleftheria Soulioti, Athanasia Tsaroucha, Alexandros Makris, Maria Koutsaki, Eirini Sklika, Argyro Mela, Panayiotis D. Megaloikonomos, Andreas F. Mavrogenis, Argyro Fassoulaki

**Affiliations:** 1 Second Department of Anesthesiology, School of Medicine, National and Kapodistrian University of Athens, 12462 Athens, Greece; 2 First Department of Anesthesiology, School of Medicine, National and Kapodistrian University of Athens, 11528 Athens, Greece; 3 Department of Anesthesiology, Asklepieion Hospital of Voula, 16673 Athens, Greece; 4 Anesthesiologist, National Emergency Aid Center, 73133 Chania, Crete, Greece; 5 First Department of Orthopaedics, School of Medicine, National and Kapodistrian University of Athens, 12462 Athens, Greece

**Keywords:** tramadol, ropivacaine, brachial plexus, interscalene block, postoperative pain management, shoulder surgery

## Abstract

*Background and objectives:* Brachial plexus block is commonly used in shoulder surgery, as it provides satisfactory surgical conditions and adequate postoperative pain control. However, there are contradictory reports regarding the addition of tramadol to the injected regional anesthetic solution. We performed a prospective randomized study to evaluate the effectiveness of tramadol as an adjuvant to ropivacaine during interscalene brachial plexus block and assess its impact on the opioid consumption and the early postoperative pain in patients that underwent shoulder surgery. *Materials and Methods:* Eighty patients scheduled for elective shoulder surgery and anesthesia via interscalene brachial plexus block were randomly divided into two groups. In group A (*n* = 40), a solution of 40 mL of ropivacaine 0.5% and 2 mL (100 mg) of tramadol was administered during the brachial plexus block, while in group B (*n* = 40), a solution of 40 mL of ropivacaine 0.5% and 2 mL NaCl 0.9% (placebo) was administered. The effectiveness and duration of sensory and motor blocks were recorded in both groups. The sensory block was assessed recording the loss of sensation to pin prick test over the skin distribution of the axillary, radial, and musculocutaneous nerves. The motor block was assessed using the modified 3-point Bromage score (0–2 points). Cumulative morphine consumption and pain, using the Visual Analog Scale (VAS), were evaluated in both groups at 2, 4, 8, and 24 h after surgery. *Results:* Sensory block onset was achieved earlier in group A than in group B (5.21 ± 3.15 minutes (min) vs. 7.1 ± 4.51 min, *p* = 0.029). The motor block onset was similar between the two groups (13.08 ± 6.23 min vs. 13.28 ± 6.59 min; *p* = 0.932). The duration of the sensory block was longer in group A as compared to group B (13 ± 2.3 h vs. 12 ± 2.8 h; *p* = 0.013). The duration of the motor block did not present any difference between the groups (10 ± 2.2 h vs. 10 ± 2.8 h; *p* = 0.308). Differences in morphine administration were not significant at 2, 4, and 8 h, however, morphine consumption was found to be decreased in group A 24 h postoperatively A (*p* = 0.04). The values of VAS were similar at 2, 4, and 8 h, however, they were lower in group A at 24 h (*p* < 0.013). *Conclusions:* Combined regional administration of tramadol and ropivacaine during interscalene brachial plexus block improves the time of onset and the duration of the sensory block, while it is associated with reduced morphine consumption during the first 24 h after shoulder surgery.

## 1. Introduction

Postoperative pain management is important after shoulder surgery for analgesia and optimal patient rehabilitation. The interscalene approach to the brachial plexus is a commonly used regional anesthesia technique for shoulder surgery that provides satisfactory surgical conditions and adequate postoperative pain control. Compared to general anesthesia, peripheral nerve blocks offer several advantages such as lower opioid consumption, decreased incidence of postoperative nausea and vomiting, lower length of stay in the postanesthesia care unit, possible shorter duration of hospitalization, and increased patient satisfaction [1,2,3,4]. A previous study also showed that performance of brachial plexus block compared to general anesthesia does not even delay the time to surgical incision [4]. In this regard, the time required for the onset of sensory and motor blocks is important, and several adjuvants have been administered to enhance the brachial plexus block onset and duration.

Tramadol is a weak μ-opioid receptor (MOR) agonist with its active metabolite (desmetramdol) having a 700-fold higher affinity for the MOR receptor but not penetrating the central nervous system (CNS) [5]; desmetramdol inhibits norepinephrine and serotonin reuptake and also targets some ligand-gated ion channels and G-protein coupled receptors (GPCR) [6]. The antinociceptive effect of tramadol involves its effect on the µ_1_ of the MOR but also on other proteins such as GPCR, ion channels, and monoamine transporters [7].

Several studies, including two systematic reviews [8,9], have concluded with contradictory results and recommendations regarding the addition of tramadol to the local anesthetic solution injected for brachial plexus block of different regional anesthesia approaches. To confirm or negate these conflicting reports, we performed this prospective randomized study to evaluate the effectiveness of tramadol as adjuvant to ropivacaine for interscalene brachial plexus block on the opioid consumption and early postoperative pain in patients who underwent shoulder surgery. Our hypothesis was that tramadol, when added to the local anesthetic solution injected for brachial plexus block, may improve analgesia after shoulder surgery.

## 2. Materials and Methods

**Figure 1:**
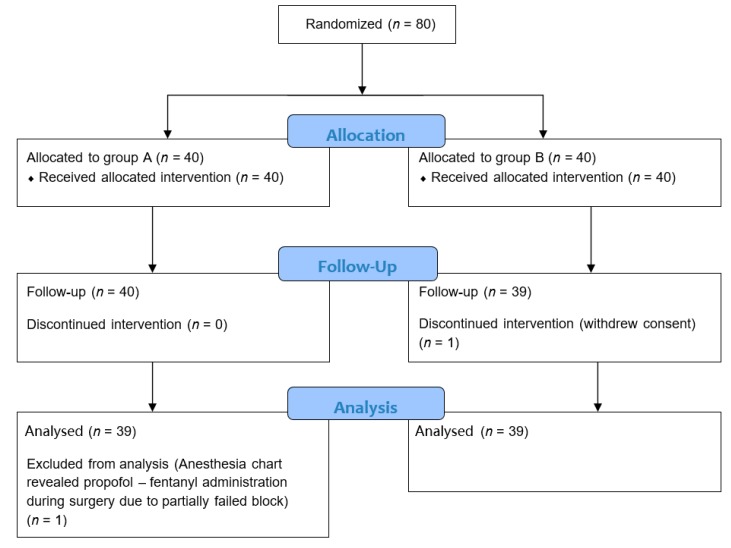
Flow diagram of the study.

Patients scheduled for elective and trauma shoulder surgery were enrolled in the study; patients older than 79 years or younger than 18 years, with a physical status according to the American Society of Anesthesiologists (ASA) of more than III, suffering from diabetes mellitus or nervous system disease, consuming opioids or other analgesics, reporting allergy or intolerance to local anesthetics, being under antiplatelet and anticoagulation therapy, those with chronic (>6 months) shoulder pain, and those refusing to participate in the study were excluded. Overall, 80 patients were included in the study; one patient withdrew consent during the study and another, after reevaluation of the anesthesia chart, was excluded from further analysis due to additional anesthetic drugs administration (Figure 1). This left 78 patients to be included in this study; there were 40 patients with a humeral head and/or neck treated with an open hemi- or reverse arthroplasty of the shoulder, 36 patients with a rotator cuff tear treated with an arthroscopic rotator cuff repair with sutures and anchors, and 2 patients with shoulder arthritis treated with an open hemi- or reverse arthroplasty of the shoulder (Table 1). No patient had any history or symptoms of cervical radiculopathy at presentation. All patients gave written informed consent for their data to be used in this study. The study was approved by the Institutional Review Board/Committee of the authors’ institution (Φ-1/28-03-2013; approved on 28 March 2013), it was registered in the ClinicalTrials.gov registry with the identification number NCT02182752, and it adheres to CONSORT guidelines.

The patients were randomly allocated into two groups using a sealed envelope method; 80 opaque envelopes were prepared and coded as group A (tramadol adjuvant administration) and group B (placebo adjuvant administration). An envelope indicating the group assignment was opened for each patient in the operating room before preparing the local anesthetic solution for the interscalene block. The envelopes were opened and the anesthetic solution for the interscalene brachial plexus block was prepared by an anesthesiology nurse that was blinded and not involved in the study.

In all patients, the same regional anesthesia technique was used. The patients were assessed the evening before surgery and the visual analogue scale (VAS) pain score with a 0–100 mm line indicating pain intensity (0 indicated no pain; 100 mm indicated the worst excruciating pain) was explained to them. Premedication was omitted. In the operating room, monitoring included pulse oximetry, electrocardiogram, and noninvasive blood pressure measurement using an S/5 Light Monitor (Datex-Ohmeda, Madison, WI, USA). An 18-gauge intravenous cannula was inserted in a peripheral vein of the opposite hand of the side to be operated on. Ringer’s lactate infusion was administered at a rate of 5 mL/kg/h and a face mask providing supplemental oxygen (6 L/min) was applied. Metoclopramide 10 mg, ranitidine 50 mg, droperidol 0.75 mg, and fentanyl 2 µg/kg were administered intravenously before starting the brachial plexus block procedure. The brachial plexus block was performed using the modified lateral technique as described by Borgeat [10].

With the patient in the supine position and the head turned away from the side to be blocked, the following superficial landmarks were marked: the lateral edge of the sternocleidomastoid muscle, the interscalene groove (IG) between the anterior and middle scalene muscles, the clavicle, the cricoid cartilage, and its lateral projection towards the IG. After skin antisepsis with chlorexidine 0.5% in alcohol 70%, local skin infiltration with 2–3 mL of lidocaine 2% was done, and a puncture was made 0.5 cm caudal to the cricoid cartilage in the IG with the needle tip directed caudally in a direction depending on the plane of the interscalene groove. Puncture was performed with an insulated nerve-stimulating 22G needle (Stimuplex, B. Brown, Melsungen, Germany) connected to a nerve stimulator (MultiStim SENSOR PAJUNK®, Norcross, GA, USA). A motor response in the posterior and dorsal part of the superior or middle trunk of the brachial plexus was evaluated, and 40 mL of ropivacaine 0.5% were injected slowly, aspirating every 5 mL. In group A, a volume of 2 mL (100 mg) of tramadol and in group B, equal volume of NaCl 0.9% were added to the local anesthetic solution. Perineural use of tramadol was off label. All blocks were performed by the same anesthesiologist with more than seven years of experience in regional anesthesia techniques.

The start time for clinical assessments was after completion of the injection of the local anesthetic solution (time 0). The sensory block was assessed by loss of sensation to pin prick test over the skin areas supplied by the axillary, radial, and musculocutaneous nerves (skin over the deltoid muscle, upper third of posterior forearm, and lateral forearm, respectively). The motor block was assessed using a 3-point modified Bromage score [11], where 0 points indicate no motor block at full extension and flexion of all upper extremity joints, 1 point indicates decreased motor strength with ability to move only the fingers, and 2 points indicate complete motor block with inability to move the elbow, wrist, and fingers. The anesthesiologist assessing all study parameters was blinded to the patients’ group allocation and the drugs used, as these were administered by the anesthesiology nurse. Thirty minutes after the end of the anesthetic solution injection, if there were signs of incomplete or failed motor and/or sensory block, supplementary analgesics were given or the block was converted to general anesthesia and the case was excluded from further analysis for this study. When the surgical operation was completed, the patients were transferred to the postanesthesia care unit (PACU), and then to the orthopaedics wards. Systolic and diastolic arterial pressure (SAP, DAP, respectively), heart rate (HR), and pulse oximeter oxygen saturation (SpO_2_) were recorded before and after the local anesthetic solution injection, on the onset of the sensory block, and on the onset of the motor block. The patients were also observed intra- and postoperatively for appearance of Horner syndrome, hoarseness, and nausea/vomiting. The anesthesiologist recording all measurements was also blinded to the patients’ group allocation.

Pain was assessed at 2, 4, 8, and 24 h postoperatively, using the VAS score. All patients received 1 g of intravenous paracetamol 8-hourly postoperatively. Morphine 0.1 mg/kg was administered subcutaneously as rescue analgesia if VAS was ≥40 mm or upon patient demand. In case of nonadequate pain relief with morphine within 4 h of morphine injection, diclophenac 75 mg was slowly injected intravenously within 30 minutes, diluted in 100 mL of 0.9% NaCl. We evaluated the onset and duration times of the sensory and motor blocks, the cumulative morphine consumption, and VAS scores at 2, 4, 8, and 24 h postoperatively.

Sample size estimation using power analysis showed that a total of 35 patients were necessary for each group to detect a clinically relevant reduction of analgesics requirements by 30% with a power of 0.8 and level of significance of 5%. However, to control for possible patients’ dropouts, a total of 80 patients were recruited, 40 for each group. The differences in patients’ clinical characteristics between the two treatment groups (age, body mass index (BMI), and duration of surgery) were evaluated with the Mann–Whitney test for non-normally distributed responses. The differences in patients’ clinical signs between the two treatment groups (SAP, DAP, HR, and SpO_2_) were evaluated with the Mann–Whitney test for normally distributed responses; for categorical variables, numbers and percentages were reported, and the differences between the two groups were compared with the χ^2^ test or the Fisher’s exact test. All results were presented in frequency tables. Both groups of patients were comparable regarding patient characteristics, ASA physical status type, and type and duration of surgery (Table 1). Overall group differences in VAS, cumulative morphine, and diclophenac consumption were assessed with the repeated ANOVA measures, and the differences in each time point were assessed with the Wilcoxon test. Statistical analysis was done with the SPSS 11.0v. (SPSS Inc., Chicago, IL, USA).

## 3. Results

Morphine consumption measured at the different time points differed overall during the 24 h; the difference was significant only at 24 h postoperatively (*z* = −2.059; *p* = 0.04). Eight patients in group A asked for rescue analgesia (morphine) compared to 17 patients in group B (χ^2^ = 4.768, df = 1, *p* = 0.029). One patient in each group, in addition to morphine, received diclophenac for postoperative pain. VAS scores recorded for the overall 24 h postoperatively in group A were lower than the VAS scores reported in group B; however, the difference was significant only at 24 h postoperatively (*z* = −2.482, *p* = 0.013) (Table 2).

Sensory block onset was at 5.21 ± 3.15 minutes in group A and at 7.10 ± 4.51 minutes in group B (*z* = −2.188, *p* = 0.029). Motor block onset was at 13.08 ± 6.23 minutes in group A and at 13.28 ± 6.59 min in group B; the differences were not statistically significant (*z* = −0.085, *p* = 0.932). The duration of the sensory block was 13 ± 2.3 h in group A and 12 ± 2.8 h in group B; the difference was statistically significant (*z* = 2.475, *p* = 0.013). The duration of the motor block was 10 ± 2.2 h in group A and 10 ± 2.8 h in group B; the difference was not statistically significant (*z* = 1.019, *p* = 0.308) (Table 3).

Hemodynamics and SpO_2_ did not differ between the groups after the onset of the nerve block (Table 4). Six patients in the tramadol group and five patients in the control group experienced Horner’s syndrome (χ^2^ = 0.106, df = 1, *p* = 0.745). Hoarseness was observed in five patients in group A and in five patients in group B (χ^2^ = 0.147, df = 1, *p* = 0.632). No patient experienced postoperative nausea/vomiting.

## 4. Discussion

Adjuvants to local anesthetic solutions for brachial plexus block are commonly used because they may enhance the onset and duration of the sensory block and decrease postoperative analgesic requirements. The results of the present randomized controlled study showed that the addition of 100 mg tramadol to 0.5% ropivacaine for interscalene brachial plexus block results in lower pain scores and reduced cumulative morphine consumption 24 h after shoulder surgery.

In a double-blind randomized clinical study, Nagpal et al. [12] reported that 100 mg of tramadol injected perineurally along with 0.5% bupivacaine solution for supraclavicular brachial plexus block resulted in a faster onset of both sensory and motor blocks, prolonged duration of the motor block, and delayed demands for rescue analgesia compared to bupivacaine alone or to bupivacaine plus tramadol intravenous injection [12]. Another randomized controlled study with patients scheduled for carpal tunnel release performed under axillary brachial plexus block [13] reported that the addition of 100 mg tramadol to the 0.75% ropivacaine solution enhanced the onset of the nerve block compared to the control group, and prolonged anesthesia and analgesia similarly to that obtained with clonidine or sufentanil as adjuvants but with fewer side effects [13]. Other authors reported that tramadol 1.5 mg/kg administered perineurally or intramuscularly along with 0.4 mL/Kg of 0.5% levobupivacaine for middle interscalene block was associated with longer duration of analgesia compared to placebo (0.9% NaCl) [14]. However, tramadol administered by the perineural route also produced a longer duration of analgesia than tramadol administered intramuscularly. The onset time of the nerve block was not prolonged by the perineural or by the intramuscular route of administration of tramadol, and did not differ from the onset time observed in the control group [14].

Kapral et al. [15], in a randomized, controlled double-blind study showed that the addition of 100 mg tramadol to mepivacaine 1% for axillary plexus block prolonged sensory and motor blocks when compared with mepivacaine alone or mepivacaine and intravenous administration of 100 mg of tramadol; the onsets of the sensory and motor blocks did not differ between the groups. Robaux et al. [16] added 0.9% sodium chloride compared to 40, 100, or 200 mg tramadol to 40 mL of 1.5% mepivacaine injection for axillary nerve block; fewer patients asked for postoperative analgesia in the tramadol groups compared to the controls in a dose-dependent pattern. However, this study failed to show an effect on the onset and duration of the sensory and motor blocks [16]. Kaabachi et al. [17] reported that 200 mg tramadol prolonged the duration of the sensory block and postoperative analgesia when added to 1.5% lidocaine plus 1/200,000 epinephrine solution for axillary nerve block for hand surgical operations. However, the onset of the sensory block was also prolonged in the group that received 200 mg tramadol perineurally, which is a limitation of the benefit of prolonged analgesia [17].

A dose-dependent effect of tramadol has been reported [18,19,20]. In a study, 100 mg tramadol added as adjuvant to bupivacaine for supraclavicular brachial plexus block enhanced the onset and duration of both sensory and motor blocks and decreased the postoperative analgesic requirements compared to intravenous tramadol or placebo administration [18]. Other authors reported that 50 mg tramadol added to 40 mL 0.375% ropivacaine for axillary nerve block in patients undergoing forearm and hand surgery was associated with a more rapid onset of the block, a shorter duration of the sensory and motor blocks, and a longer duration of analgesia when compared with the control group or with 50 mg ketamine injected perineurally as adjuvant [19]. The longer duration of analgesia along with the shortest duration of the sensory and motor blocks reported by the authors in the tramadol group sound inconsistent and are difficult to explain. Other investigators who used 0.75% ropivacaine to perform axillary nerve block found that perineural injection of 100 mg tramadol had no effect on the speed of onset, the duration of sensory and motor blocks, or the duration of postoperative analgesia [19]. Similarly, in another study, tramadol 1.5 mg/kg added to 1.5% prilocaine did not enhance the onset nor prolong the duration of analgesia [21]. Tramadol 100 mg added to a solution of local anesthetics comprising 30 mL of 0.5% levobupivacaine and 10 mL of 2% lidocaine administered for axillary nerve block had no effect on the onset or duration of the sensory block or postoperative analgesia, except that the adjuvant delayed the motor block onset on the median and ulnar nerves but not on the other nerves of the upper extremity [22].

Two systematic reviews also ended up with different conclusions and recommendations [8,9]. The first, based on eight studies, suggested that patients undergoing surgery under brachial plexus block will not benefit from tramadol added as adjuvant as there is lack of effectiveness to prolong analgesia; additionally, the potential of neurotoxicity of tramadol when injected perineurally cannot be excluded [8]. In contrast, the second review and meta-analysis that included 16 randomized control studies and 751 patients showed that 100 mg but not 50 mg tramadol as adjuvant prolonged the duration of the sensory and motor blocks, and eventually prolonged postoperative analgesia with no impact on adverse effects [9]. The onset of sensory and motor blocks was also enhanced [9]. In general, the overall value of tramadol as adjuvant to the local anesthetics to enhance brachial plexus block remains uncertain, and the potential of neurotoxicity cannot be entirely excluded. Studies investigating the coadministration of tramadol to local anesthetics in brachial plexus block vary with respect to the brachial plexus approach (interscalene, axillary, supra- or infraclavicular), the method to determine the injection site (ultrasound or with a peripheral nerve stimulator), the different local anesthetic, and the dose and route of tramadol administration (perineural, intramuscular, or intravenous). These may be some of the reasons for yielding contradictory results.

Strengths of the study are the randomization and blinding, and low number of patients’ dropouts. The fact that ultrasonographic detection of diaphragm paralysis, which is the most distressing complication when a high volume of local anesthetic is used to perform brachial plexus block, was abandoned is a major limitation of the present study; however, no patient included in this study experienced any ventilation disturbances intraoperatively. This emphasizes the safety of the anesthetic regimen used in this study for intra- and postoperative anesthesia in these patients. The use of ultrasound guidance for the detection of the end of the needle was abandoned on purpose because it was reported to have no impact on the time of duration of the sensory block as compared to ultrasound guidance or dual guidance [23]. Additionally, the procedures were performed by the same anesthesiologist which compensates for a decreased accuracy regarding the site of injection and therefore an increased variability of drug dispersion during the nerve block. The use of the high volume of 40 mL of local anesthetic was responsible for the impressive high success rate in successful blocks according to the current literature, because reduction of volume to 20 mL results in the necessity of conversion to general anesthesia because of insufficient block in 58.54% of cases where only perineural stimulator is used [24]. Although a high volume of ropivacaine (40 mL) was used, only 11 patients experienced Horner’s syndrome, what is relatively low as compared to the current literature [25].

## 5. Conclusions

In conclusion, perineural combined administration of 100 mg tramadol and ropivacaine during interscalene brachial plexus block enhances the time of onset and the duration of the sensory block, and is associated with reduced morphine consumption during the first 24 h after shoulder surgery. The low rate of incidence of complications is the strong aspect of this article, as it is important for everyday practitioners constituting the safety of the anesthetic regimen for the improvement of the postoperative analgesia with the addition of tramadol to ropivacaine.

## Figures and Tables

**Table 1:** Demographic and procedure data. Data is presented as mean values ± SD, or absolute numbers.

Variables	Group A (*n* = 39)	Group B (*n* = 39)	Statistics
Age (years)	63 ± 11	67 ± 11	*z* = 1.59 *p* = 0.112
ΒΜΙ	27.6 ± 4.3	26.9 ± 4.5	*z* = 0.87 *p* = 0.382
ASA (I/II/III)	12/24/3	10/25/4	χ^2^ = 0.345 *p* = 0.842
Type of surgery			
• Shoulder fracture	21	19	51.282%
• Rotator cuff injury	17	19	46.153%
• Shoulder arthritis	1	1	2.564%
Duration (min)	76 ± 35	77 ± 38	*z* = 0.09 *p* = 0.928

BMI, Body Mass Index; ASA, American Society of Anesthesiologists physical status classification; min, minutes.

**Table 2:** Morphine consumption (mg) and VAS scores (mm) at 2, 4, 8, and 24 h in group A (tramadol) and group B (placebo). Data is presented as mean values ± SD, and minimum–maximum values.

Variables	Group A (*n* = 39)	Group B (*n* = 39)	Statistics
**Morphine consumption**			
2 h	0	0	*z* = 0 *p* = 1
4 h	0	0	*z* = 0 *p* = 1
8 h	0	0.34 ± 1,49	*z* = −1.423 *p* = 0.155
24 h	1.55 ± 3.12 0–9.5	3.42 ± 4.2 0–13.6	*z* = −2.059 *p* = 0.04
**VAS scores**			
2 h	0	0	*z* = 0 *p* = 1
4 h	0	0	*z* = 0 *p* = 1
8 h	0	0.15 ± 0.71 0–40	z = −1.423 *p* = 0.155
24 h	0.28 ± 0.76 0–30	0.92 ± 1.31 0–40	*z* = −2.482 *p* = 0.013

**Table 3:** Onset (minutes) and duration (hours) of the sensory and motor blocks in group A (tramadol) and group B (placebo) at the end of injection as well as at the onset of the sensory and motor blocks. Data is presented as mean values ± SD, and minimum–maximum values.

**Onset of the sensory block**	
Group A	5.21 ± 3.15 1–15
Group B	7.10 ± 4.51 1–25
Statistics	*z* = −2.188 *p* = 0.029
**Onset of the motor block**	
Group A	13.08 ± 6.23 5–32
Group B	13.28 ± 6.59 3–30
Statistics	*z* = −0.085 *p* = 0.932
**Duration of the sensory block**	
Group A	13 ± 2.3 8–17
Group B	12 ± 2.8 6.5–18
Statistics	*z* = 2.475 *p* = 0.013
**Duration of the motor block**	
Group A	10 ± 2.25 6–15
Group B	10 ± 2.8 4–16
Statistics	*z* = 1.019 *p* = 0.308

**Table 4:** Systolic (SAP, mm Hg) and diastolic (DAP, mm Hg) arterial pressure, heart rate (HR, beats per minute), and arterial oxygen saturation (SpO_2_ %) in group A (tramadol) and group B (placebo) before injection, at the end of injection, and at the onset of the sensory and motor blocks. Data is presented as mean values ± SD, and minimum–maximum values.

**SAP**	**Group A**	149 ± 16.8 117–188	145 ± 18.1 100–172	144 ± 19.2 102–180	140 ± 15.9 100–165
	**Group B**	150 ± 17.9 112–180	149 ± 19.8 105–180	142 ± 16.9 110–175	143 ± 17.3 111–179
	**Statistics**	*z* = 0.395 *p* = 0.693	*z* = 1.05 *p* = 0.294	*z* = 0.0.42 *p* = 0.674	*z* = 0.551 *p* = 0.582
**DAP**	**Group A**	80 ± 9.5 58–97	80 ± 9.1 56–101	79 ± 8.8 56–93	79 ± 10 56–94
	**Group B**	81 ± 9.3 60–100	82 ± 9.4 66–99	79 ± 9.2 60–98	79 ± 10 60–99
	**Statistics**	*z* = 0.381 *p* = 0.703	*z* = 0.736 *p* = 0.461	*z* = 0.120 *p* = 0.904	*z* = 0.115 *p* = 0.908
**HR**	**Group A**	78 ± 14.2 57–117	78 ± 14.5 55–110	77 ± 13.7 58–107	77 ± 13.1 60–106
	**Group B**	82 ± 11.2 60–105	81 ± 10.7 61–102	80 ± 11.2 57–105	80 ± 11 57–104
	**Statistics**	*z* = 1.231 *p* = 0.218	*z* = 1.031 *p* = 0.302	*z* = 0.926 *p* = 0.354	*z* = 1.371 *p* = 0.170
**SpO_2_**	**Group A**	99 ± 1.4 94–100	99 ± 1.3 94–100	99 ± 1.3 95–100	99 ± 1.1 96–100
	**Group B**	99 ± 1.56 94–100	99 ± 1.5 94–100	99 ± 1.3 95–100	99 ± 1.3 95–100
	**Statistics**	*z* = 0.311 *p* = 0.755	*z* = 0.168 *p* = 0.867	*z* = 0.686 *p* = 0.493	*z* = 1.048 *p* = 0.295
